# Bioactive glass S53P4 cream kills ESKAPE panel multidrug resistant pathogens and *Staphylococcus aureus* biofilms

**DOI:** 10.3389/fphar.2026.1768338

**Published:** 2026-05-04

**Authors:** Deeksha Rajkumar, Adrian Stiller, Payal P. S. Balraadjsing, Leena Hupa, Sebastian A. J. Zaat

**Affiliations:** 1 Department of Medical Microbiology and Infection Prevention, Amsterdam Institute for Immunology and Infectious Diseases, Amsterdam UMC, University of Amsterdam, Amsterdam, Netherlands; 2 Laboratory of Molecular Science and Engineering, Åbo Akademi, Turku, Finland

**Keywords:** antimicrobial resistance (AMR), bioactive glass, icp-oes, multidrug-resistant (MDR), orthopedic implant-associated infection, *Staphylococcus aureus*, titanium

## Abstract

**Introduction:**

Orthopedic implant-associated infections, predominantly caused by S. aureus, pose significant challenges due to biofilm formation and antibiotic resistance. Bioactive Glass (BAG) S53P4 is a unique material with antimicrobial and bone regenerative properties. We aimed to characterize a novel BAG S53P4 cream, consisting of BAG powder and a binder, for its capacity to kill *Staphylococcus aureus* in suspension and biofilms in the absence or presence of titanium implant material. Since the BAG antimicrobial activity depends on ions eluted, we also analyzed the eluates of the cream and of powder and binder.

**Methods:**

BAG cream, BAG powder, and binder were evaluated for antimicrobial activity against planktonic *S. aureus* in the presence or absence of titanium implant material, and against *S. aureus* biofilms. Eluates collected at different time points were tested against a panel of bacterial and fungal pathogens. Elemental ion release and pH changes were measured over time. Time-kill and biofilm assays were performed. Additionally, the applicability and antimicrobial efficacy of BAG cream were evaluated in a cadaver mouse bone defect model.

**Results:**

The BAG cream and BAG powder applied to titanium implant material, as well as their respective eluates eradicated planktonic S. aureus. Elemental release from BAG cream and powder showed time-dependent shifts in levels of silicon, sodium, calcium and phosphorous together with stable alkaline pH levels, reflecting continuous ion release from the glass network and concurrent precipitation of calcium phosphate and silica phases. BAG cream and powder eluates collected as early as at 2 h were highly effective against S. aureus, the ESKAPE panel of multidrug resistant pathogens, colistin-resistant Escherichia coli and Cutibacterium acnes, and against the fungi *Candidozyma auris* and Candida albicans. The eluates displayed time-dependent bactericidal activity with significant bacterial killing starting already at 30 min and increasing with longer exposure times. Moreover, significant reduction in *S. aureus* biofilm was observed with the cream and powder eluates. BAG cream was easy to apply to the bone defect of a cadaver mouse using a syringe and it effectively prevented S. aureus growth.

**Conclusion:**

These findings show the potential of BAG cream as an innovative application form of BAG S53P4 offering a promising approach against orthopedic implant-associated infections.

## 1 Introduction

Orthopedic implant-associated infections (OIAIs) represent a significant challenge in medical practice, often leading to severe complications in patients. The success of orthopedic implants in restoring bone function and enhancing intended implant integration can be hampered by infection (Barros et al., 2019; Kumar and Roger, 2019), as pathogens can induce persistent inflammation and osteolysis. *Staphylococcus aureus* is the most common pathogen causing OIAI (Osmon et al., 2013), and its ability to form biofilms on implant surfaces further complicates treatment by enhancing antibiotic resistance (Koo et al., 2017; Zimmerli et al., 2004). Multidrug resistant pathogens, including members of the ESKAPE group (*Enterococcus faecium*, *S. aureus, Klebsiella pneumoniae*, *Acinetobacter baumannii*, *Pseudomonas aeruginosa*, and *Enterobacter cloacae*) as well as colistin-resistant *Escherichia coli* are also increasingly associated with implant infections and pose additional treatment challenges (de Breij et al., 2018). Although less common in OIAIs, fungal pathogens such as *Candida albicans* and *Candidozyma auris,* formerly known as *Candida auris,* are emerging as concerns, particularly in immunocompromised patients (Kaur and Nobile, 2023; Liu et al., 2024; Pallotta et al., 2023). The World Health Organization (WHO) has identified these bacterial and fungal pathogens as critical priorities for the development of new antimicrobial strategies (WHO, 2017; WHO, 2022). Anaerobic skin-associated bacteria like *Cutibacterium acnes*, often introduced during surgery, have also been implicated in low-grade or delayed implant infections (Boisrenoult, 2018). The growing prevalence of antimicrobial-resistant microorganisms and the persisting challenge in treating biofilms underscore the critical need for more effective non-antibiotic antimicrobial strategies. Bioactive Glass S53P4 cream represents one such promising novel agent.

Bioactive glasses are a unique class of biomaterials that offer antimicrobial, osteoconductive, and osteostimulatory properties beneficial for infection control and bone regeneration, since it can also bond with bone and soft tissues (Hoppe et al., 2011; Miguez-Pacheco et al., 2015). Various formulations, including 45S5 and doped variants, have been tailored for bone infection management and regeneration (Hench, 2006). Among them, Bioactive glass (BAG) S53P4 is considered a valuable adjunct to antibiotics in treating OIAIs (Lindfors et al., 2010). Upon contact with body fluids, BAG S53P4 releases sodium, calcium and phosphate ions and soluble silica species, creating an alkaline, high-osmotic environment that is considered to disrupt bacterial membranes and metabolism, leading to bacterial cell death (Drago et al., 2015; van Gestel et al., 2015). Additionally, these glass dissolution products have been reported to stimulate osteoblast activity and the expression of bone-related genes, thereby promoting osteogenesis (Eriksson et al., 2021; Xynos et al., 2001). The antibacterial and bone-regenerative function highlights BAG S53P4’s potential as an alternative to conventional antibiotic therapies in managing OIAIs.

BAG S53P4 can be produced in various forms, including granules (500–800 µm), powder (≤45 µm), scaffolds, and putty (Drago et al., 2014). Clinically, granules and putty are used as bone defect fillers because of their osteoconductive properties that promote bone regeneration (Drago et al., 2015), whereas preclinically scaffolds provide structural support for complex bone reconstructions (Eriksson et al., 2021). BAG powder, although not clinically used, is a promising candidate for preventing and treating OIAIs because its small particle size and large surface area promote a high initial rate of ion release. When combined with a polymeric binder, BAG powder can improve application on implant surfaces while also enhancing antibacterial activity in poorly vascularized bone sites where systemic antibiotic treatment is less effective (Maximov et al., 2021; Munukka et al., 2008). Developing BAG powder into a cream formulation for implant application can therefore significantly strengthen its practical use during orthopedic surgical procedures.

This study aimed to investigate the bactericidal and biofilm killing activity of a novel BAG S53P4 cream formulation, with BAG powder as the key antimicrobial component. The efficacy of the cream was investigated *in vitro* and in a cadaver mouse *S. aureus* bone-defect model, to assess its potential to prevent and treat OIAIs.

## 2 Materials and methods

### 2.1 Bioactive glass S53P4

Three different sample forms based on Bioactive Glass (BAG) S53P4 were used in this study: granules (500–800 µm), powder (<25 µm) and cream (Supplementary Figure S1). The cream consists of 50 wt% powder (<25 µm) and 50 wt% binder composed of different polyethylene glycol (PEG) species and glycerol. As control, the binder without BAG powder was included.

### 2.2 Microorganisms

In this study we used *S. aureus* JAR060131 (Culture Collection Of Switzerland (CCOS) number 890, Wädenswill, Switzerland) obtained from a patient with an orthopedic device related infection (Moriarty et al., 2010). Strains from the ESKAPE pathogen panel were *E. faecium* LUH15122, *S. aureus* LUH14616, *K. pneumoniae* LUH15104, *A. baumannii* RUH875, *P. aeruginosa* LUH15103, *E. cloacae* LUH15114, along with colistin-resistant *E. coli* LUH15117 (de Breij et al., 2018). Given the clinical relevance of colistin-resistant *E.coli,* it is included alongside the ESKAPE pathogens collectively referred to hereafter as ESKAPE(E). *Cutibacterium acnes*, isolated from a surgical knife (Guarch-Perez et al., 2023), and fungal strains *C. albicans* SC5314 (Hoyer et al., 2024) and *Candidozyma auris* 111 obtained from the Dutch “Foundation for Quality Assessment in Medical Laboratory Diagnostics”, were also included.

Before each experiment, bacterial and fungal cultures were prepared by incubating 1 to 3 colonies in 5 mL Tryptic Soy Broth (TSB; Difco, United States) at 37 °C and shaking (120 rpm) overnight. The next day, 100 µL of the overnight culture was sub-cultured in 5 mL fresh TSB and incubated for 3 h at 37 °C to reach mid-logarithmic growth phase. For *C. acnes*, the same procedure was followed using Todd-Hewitt Yeast (THY) broth, with anaerobic incubation at 37 °C without shaking for 3 days.

Mid-log phase cultures were washed twice with RPMI 1640 medium (20 mM HEPES, L-glutamine, without sodium bicarbonate; Sigma-Aldrich, United Kingdom), or with THY for *C. acnes*. Bacterial and fungal suspensions were then adjusted to 1 × 10^7^ CFU/mL in RPMI or THY, respectively, based on an established OD-to-CFU correlation. From this, 100 µL (1 × 10^6^ CFU) or 10 µL (1 × 10^5^ CFU) inocula were used.

### 2.3 Test methods for bactericidal activity

Two test methods were used to assess the bactericidal activity of BAG formulations: direct testing and eluate testing. In **direct testing**, 200 mg of BAG cream (containing 100 mg powder and 100 mg binder), 100 mg of BAG powder, or 100 mg of binder was evenly applied to the bottom of 24-wells plate or onto sterile Titanium Aluminum Niobium (TAN) discs (Ø13 mm × 2 mm) as titanium and its alloys are widely used in orthopedic implants. For experiments with BAG granules *versus* powder either 800, 400, 200 or 100 mg of the respective formulation was added to 5 mL test tubes. After adding 900 µL RPMI medium to the BAG formulation, the pH was measured using either a pH sensor (Eutech Instruments, Waterproof pH Testr30) or strips (MQuant, pH 0–14). Immediately following pH measurements, 100 µL of *S. aureus* JAR inoculum was added to each formulation (total volume 1 mL), and the samples were incubated at 37 °C, 120 rpm for 24 h. RPMI medium, with or without TAN discs, served as control.

For **eluate testing**, the same amounts of BAG granules, cream, powder, or binder were applied as above. After adding 1 mL of RPMI medium, samples were incubated for either 2, 4, 8, or 24 h at 37 °C and 120 rpm. Subsequently, the RPMI medium containing the eluted ions from BAG, hereafter referred to as the eluate, was collected at the respective time points, centrifuged at 20,800 RCF for 10 min to remove particles, and the pH was measured using a pH sensor or strips. Controls included RPMI medium with or without TAN discs. Subsequently, 100 µL or 10 µL of *S. aureus* JAR inoculum was added to 900 µL or 90 µL of eluate, respectively, and incubated at 37 °C, 120 rpm for 24 h.

After 24 h of exposure, incubations were plated undiluted or ten-fold serially diluted, on blood agar plates (Oxoid, United Kingdom), and incubated overnight at 37 °C for either semi-quantitative or quantitative CFU assessment. Pipette tips were changed at each dilution step.

### 2.4 Elemental solution analysis of BAG eluates

Elemental concentrations of Silicon (Si), Sodium (Na), Calcium (Ca) and Phosphorous (P) in the 2, 4, 8, and 24 h eluates of BAG cream, BAG powder and binder, and RPMI controls were analyzed using inductively coupled plasma optical emission spectrometry (ICP-OES, Optima 5300 DV; Perkin Elmer, Waltham, United States). For analysis, 400 µL of each eluate was diluted with 10 mL of ultrapure water and acidified with 200 µL of concentrated nitric acid (65 wt%; Suprapur, Merck). The instrument was calibrated with ultrapure water and certified standards (all: Spectrascan, Teknolab AS, Norway). Calibration curves were prepared using a dilution series of 0, 1, 5, and 20 ppm for all elements. The following emission lines and configurations were selected: Sodium (Na) (λ = 589.592 nm, radial), silicon (Si) (λ = 251.611 nm; axial), phosphorous (P) (λ = 214.914 nm, axial), and calcium (Ca) (λ = 317.933 nm, axial). Instrumental limits of detection (mg/L) were 0.003 (Ca), 0.025 (P), 0.21 (Na), and 0.004 (Si), corresponding to 0.080, 0.663, 5.565, and 0.106 mg/L, respectively, in the collected eluates after correcting for the dilution factor. Each ICP-OES run consisted of five scans per sample, and average elemental concentrations were calculated from five replicate measurements.

### 2.5 Broad-spectrum microbicidal activity against multidrug-resistant pathogens

Two hour eluates of BAG cream, powder, and binder (prepared as described in the test methods) were tested against the ESKAPE(E) panel, *C. acnes*, *C. albicans* and *C. auris* pathogens. In a 96-well plate, 10 µL of microbial inoculum (1 × 10^5^ CFU) was added to 90 µL of eluate and incubated for 24 h at 37 °C, 120 rpm. Incubations were then ten-fold serially diluted and plated on blood agar for CFU quantification. All conditions were tested in triplicate.

### 2.6 Time-kill assay of BAG cream and BAG powder eluates against *Staphylococcus aureus*

A time-kill assay was performed with 2 h eluates of BAG cream, powder and binder. In a 96-well plate, 10 µL of *S. aureus* JAR inoculum was exposed to 90 µL of eluate for 30 min, 2, 4, 8, and 24 h at 37 °C and 120 rpm. At each time point, triplicate incubations were ten-fold serially diluted and plated on a BA plate to determine numbers of CFU.

### 2.7 Biofilm killing activity of eluates

Eluates of BAG cream, BAG powder and binder collected at 2, 4, 8, and 24 h (prepared as described in the test methods) were used and RPMI medium incubated for the same durations served as controls. Biofilms were formed by inoculating mid-log phase *S. aureus* JAR (10^8^ CFU/mL in RPMI +2% glucose) into two 96-well plates, a “before-treatment” and “after -treatment” plate, and incubating statically for 24 h at 37 °C. After washing with PBS, biofilms from the before-treatment plate were dispersed in 100 µL of PBS by sonication to determine the initial numbers of CFU. For treatment, 100 µL eluates or control medium were added to the biofilms in the treatment plate and the plate was incubated for another 24 h. All conditions were tested in six replicates. After 24 h, biofilms were sonicated for 5 min at 37 kHz (Elma Transsonic 460) in sealed contamination-protected plates. This procedure does not affect bacterial viability. Post-sonication, the biofilms were visually checked for dispersion, and suspensions were diluted and plated on blood agar for CFU quantification.

### 2.8 Applicability and bactericidal efficacy of BAG cream in a cadaver mouse bone-defect model

To assess the practical applicability of the BAG cream in a structurally complex setting, we used a cadaver mouse bone-defect model. We introduced *S. aureus* with or without BAG cream to obtain antibacterial effect. Although this model does not reproduce active infection or host responses, it provides intact bone and soft-tissue architecture for evaluating cream application, and it allows bacterial growth in absence of treatment. Frozen C57BL/6 mouse cadavers (aged 20–28 weeks at sacrifice) were thawed at 4 °C before use (Animal Research Institute AMC (ARIA) of the Amsterdam UMC (Amsterdam, Netherlands). To analyze the bactericidal effect of BAG cream, two groups were used: infection with or without treatment, each containing 3 cadavers. The left leg of each cadaver was shaved and disinfected with 70% ethanol. A unilateral cortical bone defect was created in the femur using a drill bit (Ø0.5mm, RISystem, Lanquart, Switzerland), and 1 µL of inoculum suspension containing 2.5 × 10^4^ CFU of *S. aureus* JAR was introduced into the defect using a calibrated 10 µL pipette.

To assess applicability, BAG cream was applied directly to the defect site using a sterile syringe, ensuring uniform delivery into the cavity and surrounding area. Approximately 125 µL of BAG cream was administered per cadaver in the treatment group (n = 3), while no treatment was applied to the control group. The fascia lata and skin were closed with 6–0 vicryl sutures, and the cadavers were incubated overnight at room temperature. After incubation, femurs and surrounding soft tissues were collected, homogenized with zirconia beads (Ø2mm, BioSpec Products, 15 beads for bone and seven beads for tissue) in PBS +0.5% Tween 80 using the MagnaLyser system (Roche Diagnostics, Basel, Switzerland), followed by 10-fold serial dilution and plating for CFU quantification.

### 2.9 Scanning electron microscopy (SEM)

BAG granules and powder were mounted on aluminum SEM stubs and sputter-coated with a 4 nm platinum-palladium layer using a Leica EM ACE600 sputter coater (Microsystems, Wetzlar, Germany). Images were acquired at 3 kV using a Zeiss Sigma 300 scanning electron microscope (Zeiss, Oberkochen, Germany) at the Electron Microscopy Center Amsterdam (ECMA; Amsterdam UMC, Amsterdam, Netherlands). Each sample on the stub was examined and imaged.

### 2.10 Statistical analysis

Statistical analysis was performed using GraphPad Prism 10 (GraphPad, San Diego, CA, United States). Biofilm killing activity was analyzed by one-way ANOVA with Tukey’s *post hoc* test. Unpaired t-tests were used for the cadaver mouse bone-defect model. A *p*-value ≤0.05 was considered significant (*p = 0.01–0.05, **p = 0.001–0.01, ***p = 0.0001–0.001, ****p < 0.0001).

## 3 Results

### 3.1 Scanning electron microscopy

**FIGURE 1:**
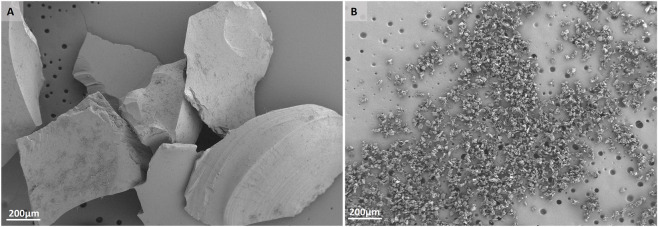
Visualization of particle sizes of BAG material using SEM, **(A)** BAG granules **(B)** BAG powder.

BAG granules and powder were visualized under SEM to assess their particle size (Figure 1). BAG powder consisted of significantly smaller particles (≤25 µm) than BAG granules (500–800 µm), resulting in a larger surface area per weight (Stoor and Frantzen, 2017).

### 3.2 BAG powder has higher bactericidal activity than granules

The bactericidal activity of BAG powder and of the clinically used BAG granules were compared using direct and eluate testing. BAG powder showed consistent bactericidal activity at all concentrations in both test methods, while granules were effective only at 800 mg/mL in eluate testing (Table 1). Both forms induced dose-dependent pH increases, with granules reaching pH 12 at 800 mg/mL in both bactericidal test methods, yet only the eluate killed *S. aureus*. At 400 mg/mL, only BAG powder and its eluate achieved complete killing of the inoculum despite identical pH values with granules, indicating that pH alone does not explain the observed activity of this concentration of powder. These results highlight the superior activity of BAG powder.

**TABLE 1:** Bactericidal activity and pH of BAG powder and granules against *Staphylococcus aureus* were evaluated using direct and eluate testing. For direct testing, pH was measured after adding RPMI to BAG, followed by bacterial exposure.

BAG	BAG powder				BAG granules			
	Direct testing		Eluate testing		Direct testing		Eluate testing	
Concentration (mg/mL)	CFU	pH	CFU	pH	CFU	pH	CFU	pH
800	-	13	-	13	+++	12	-	12
400	-	11	-	11	+++	11	+++	11
200	±	11	-	11	+++	10	+++	10
100	±	10	-	10	+++	10	+++	10
0	+++	7	+++	7	+++	7	+++	7

In eluate testing, pH was measured after 24-h elution prior to bacterial exposure. Bacterial survival after 24 h was semi-quantitatively assessed as +++ (confluent growth), ± (few colonies), or – (0 colonies) (Supplementary Figure S2).

### 3.3 BAG cream and its eluate exhibits bactericidal activity in the absence and in the presence of TAN implant materials

**FIGURE 2:**
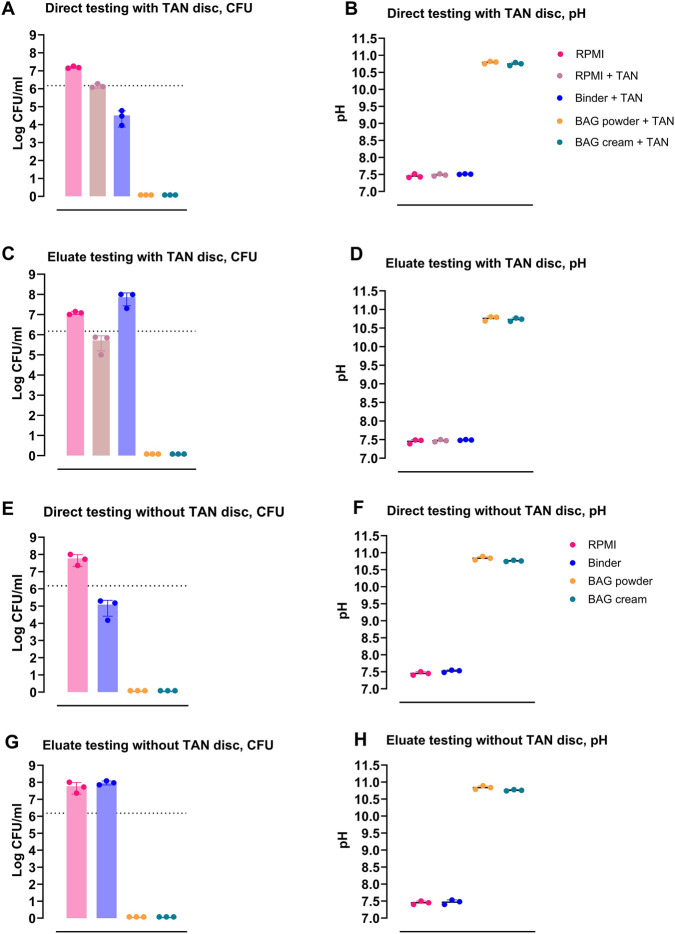
Bactericidal activity of BAG cream and the equivalent amount of BAG powder and binder in direct and 24 h eluate testing against *Staphylococcus aureus*. CFU and pH of **(A,B)** direct testing with TAN disc, **(C,D)** eluate testing with TAN disc, **(E,F)** direct testing without TAN disc and **(G,H)** eluate testing without TAN disc. Bar graphs show mean ± SD; individual data points are shown as dots, and dotted lines indicate the initial bacterial inoculum.

We assessed the bactericidal activity of BAG cream, BAG powder and binder in the absence and in the presence of TAN discs using direct testing of these materials as well as testing of their corresponding 24-h eluates. BAG cream’s consistency enabled easy application to TAN disc surfaces. Both cream, powder and their eluates completely eradicated *S. aureus* in all conditions (Figures 2A,C,E,G), with average pH values of 10.7 and 10.8 for direct and eluate testing (Figures 2B,D,F,H). The binder caused a 1-log reduction in direct testing, either with or without TAN discs (Figures 2A,E), and its eluate showed no activity (Figures 2C,G). For both direct and eluate testing of binder the pH was close to neutral with an average of 7.4–7.5 (Figures 2B,D,F,H). TAN discs alone did inhibit outgrowth of *S. aureus* but did not reduce their numbers of CFU (Figures 2A,C). The pH was not influenced by the TAN discs. Overall, BAG cream and powder showed strong, bactericidal activity and the presence of TAN did not affect their activity.

### 3.4 Element release, pH and bactericidal activity of BAG eluates collected over time

**FIGURE 3:**
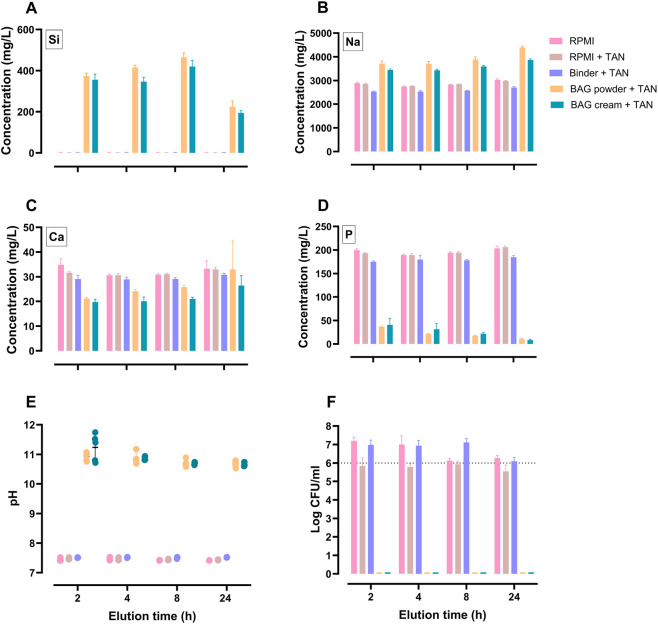
Elemental profiles, pH and bactericidal effect against *Staphylococcus aureus* of eluates from BAG cream, powder and binder obtained after 2, 4, 8 or 24 h of elution. ICP-OES quantification of **(A)** silicon (Si), **(B)** sodium (Na), **(C)** calcium (Ca) and **(D)** phosphorous (P) concentrations. **(E)** pH values of eluates of BAG cream and its equivalent components. Individual data points are depicted in dots, with mean +SD. **(F)** Bacterial survival of *Staphylococcus aureus* (log CFU/mL) after exposure to eluates. Dotted line represents the bacterial inoculum concentration. Bars indicate mean +SD.

The dissolution behavior, pH and bactericidal activity of BAG cream and powder eluates were assessed over time and compared with the control groups (RPMI medium, RPMI + TAN disc and binder + TAN disc). Elemental concentrations of silicon (Si), sodium (Na), calcium (Ca) and phosphorous (P) in their respective eluates were quantified using ICP-OES. While the control groups showed stable elemental profiles over time, the BAG cream and powder eluates exhibited pronounced time-dependent shifts (Figure 3), indicating continuous dissolution and ion release. Over the 24 h elution period, Si was not detected in any of the control groups, whereas in the BAG cream and powder eluates it increased sharply during the early phase, reaching ∼360–380 mg/L at 2 h and ∼400–470 mg/L at 8 h, before declining to ∼200–220 mg/L at 24 h (Figure 3A). Na concentration in both BAG eluates increased by approximately 30%–50% compared with the controls, rising from ∼3,000 mg/L to 3,900–4,400 mg/L (Figure 3B). Ca concentrations in the BAG eluates were ∼50% lower than the control groups at 2 h but gradually increased, returning to close to control levels by 24 h (Figure 3C). P concentration had dropped from about 180–195 mg/L in the controls to roughly 40 mg/L in both the eluates of 2 h and continued to decrease to around 10 mg/L by 24 h (Figure 3D). The eluates of the BAG cream consistently exhibited slightly lower Si, Na and Ca concentrations than those of the BAG powder throughout the elution period. The presence of binder in RPMI did not markedly affect the elemental medium composition. However, the binder + TAN disc group consistently showed slightly reduced elemental concentrations, possibly due to adsorption to the TAN discs. Presence of TAN discs did not influence the elemental levels in RPMI medium.

After 2 h elution, the pH increased markedly from an initial average values of 7.5 in the control groups to average values of 11.2 and 10.9 for BAG cream and powder eluates, respectively (Figure 3E). The pH of BAG cream and powder slightly decreased over time reaching 10.7 after 24 h of elution but remained alkaline (Figure 3E). BAG cream and powder eluates of 2 h demonstrated complete eradication of *S. aureus* and bactericidal activity maintained at longer elution times, while control groups had no bactericidal activity overtime (Figure 3F).

Upon exposure, rapid increase in Si and Na alongside a pH elevation mark the initial dissolution phase, associated with bacterial eradication with eluates of both formulations. The continuous presence, although gradually declining levels, of Si and the sustained Na release through 24 h, together with the maintenance of alkaline pH, support ongoing activity even at high area-to-volume ratio where saturation would otherwise limit dissolution. Notably, Ca showed initial depletion, suggesting precipitation or uptake, but its concentration eventually returned to near-control levels. Eluates of binder retained neutral pH, showed low ionic changes and had no antibacterial activity. This integrative, quantitative approach linking measured ion release profiles, pH, and direct CFU quantification provides strong evidence that the alkaline pH and the, rapid and sustained generation of antimicrobial dissolution products are the principal drivers of the potent bactericidal performances of BAG S53P4 cream and powder.

### 3.5 Eluates of BAG cream and BAG powder show broad-spectrum microbicidal activity against multidrug-resistant pathogens

**FIGURE 4:**
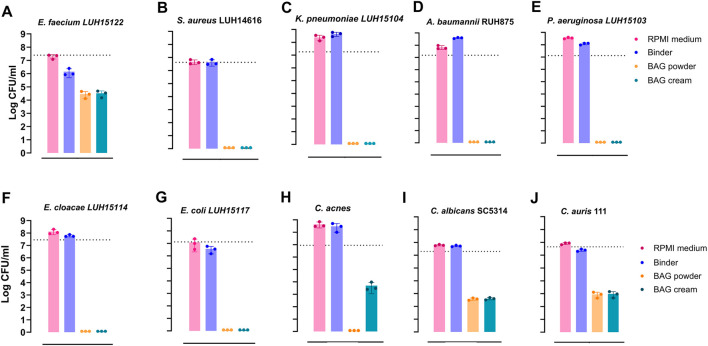
BAG cream and powder eluates show broad-spectrum microbicidal activity against multidrug-resistant bacterial and fungal pathogens. **(A–G)** ESKAPE(E) pathogens, **(H)** *C. acnes* **(I)** *C. albicans* and **(J)** *C. auris*. Microorganisms were exposed to 2 h eluates of BAG cream, powder and binder for 24 h. RPMI medium served as control. Dotted lines represent the microbial inoculum concentration. Individual data points are depicted in dots. Bars indicate mean +SD.

The broad-spectrum microbicidal activity of BAG powder, cream and binder against multi-drug resistant ESKAPE(E) pathogens, *C. acnes* and fungal pathogens was assessed using 2 h eluates. BAG cream and powder eluates completely eradicated bacterial pathogens *S. aureus* LUH14616 (Figure 4B), *K. pneumoniae* LUH15104 (Figure 4C), *A. baumannii* RUH875 (Figure 4D), *P. aeruginosa* LUH15103 (Figure 4E), *E. cloacae* LUH15114 (Figure 4F), and colistin-resistant *E. coli* LUH15117 (Figure 4G). Both cream and powder eluates caused a 3-log reduction in *E. faecium* LUH15122 (Figure 4A), while for *C. acnes*, powder eluates achieved complete killing and cream eluates caused a 3-log reduction (Figure 4H). This difference may reflect the lower ion release from cream due to the presence of binder. Antifungal assessment showed a 3-log reduction of CFU numbers of *C. albicans* SC5314 (Figure 4I) and a 2-log reduction of *C. auris* 111 CFU (Figure 4J) by both BAG cream and powder eluates. Binder eluates exhibited slight bactericidal activity against *E. faecium* LUH15122 (Figure 4A) and no antimicrobial effects on other test strains indicating that the binder itself does not contribute to microbicidal effects.

### 3.6 Time-kill analysis of BAG cream and BAG powder eluates against *Staphylococcus aureus*

**FIGURE 5:**
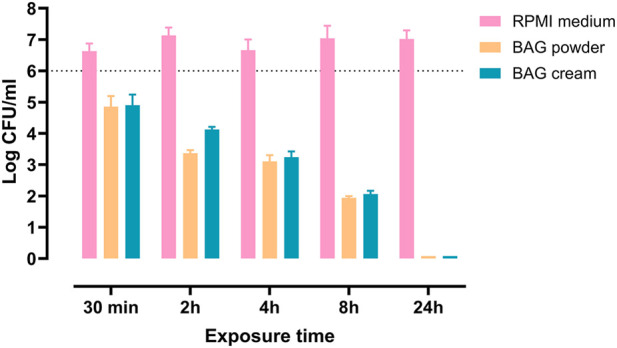
Time kill of *Staphylococcus aureus* using 2 h eluates of BAG cream and powder, with RPMI medium as control. Bars indicate mean +SD. The dotted line represents the bacterial inoculum.

To further analyze the bactericidal characteristics of the eluates, we assessed the speed of killing of *S. aureus* by BAG eluates. A time-kill assay was performed using BAG cream and powder eluates collected at 2 h, to which *S. aureus* was exposed for either 30 min or 2, 4, 8 or 24 h. The average pH values of the BAG cream and powder eluates was 10.6 and 10.7 respectively. Both eluates caused a gradual decrease in bacterial numbers over time, starting with 1-log reduction at 2 h exposure, reaching a 5-log reduction by 8 h and achieving complete eradication by 24 h (Figure 5).

### 3.7 Biofilm killing activity of eluates of BAG cream and its components

**FIGURE 6:**
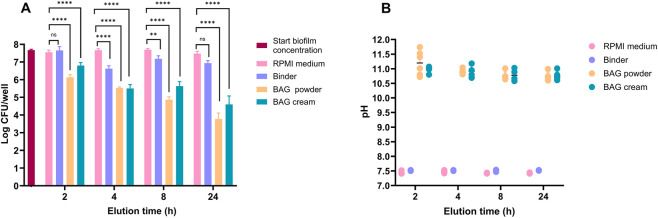
Killing of *Staphylococcus aureus* in biofilms by eluates obtained after increasing elution time. **(A)** Biofilm killing by eluates of BAG cream and its components. Bars indicate mean +SD. **(B)** The pH values of eluates of BAG cream and their components measured after 2, 4, 8 and 24 h. Individual data points are depicted in dots.

The biofilm killing activity of BAG cream and its constituents, BAG powder and binder, was assessed using eluates collected at 2, 4, 8 and 24 h. For our earlier experiments with planktonic bacteria above we used 2 h eluates, since even at this earliest time point the eluate demonstrated maximal bactericidal activity. However, since *S. aureus* biofilms are more resilient against antimicrobials, and since BAG elution continues over time, later eluates were analyzed for their effect on the biofilms as well. While 2-h eluates caused modest biofilm killing, the bactericidal effect was significantly stronger for eluates obtained after longer elution, and was highest for eluates obtained after 24 h of elution, which caused a 3-4 log reduction of numbers of CFU of the biofilms (Figure 6A). This effect correlated with alkaline eluates with average pH values ranging from 10.9–11.2 for BAG cream and powder eluates (Figure 6B). Binder eluates exhibited minimal biofilm killing activity, correlating with their near-neutral pH of 7.5 on average (Figure 6B).

### 3.8 Applicability and bactericidal activity of BAG cream in a cadaver mouse bone-defect model

**FIGURE 7:**
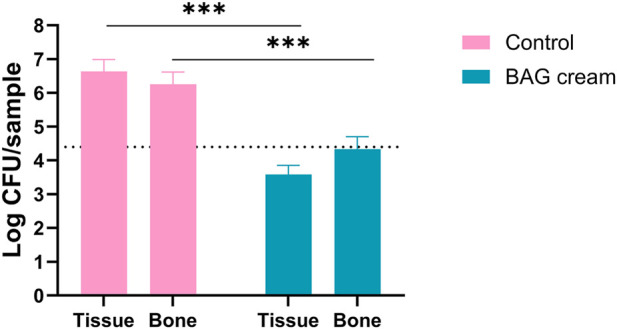
BAG cream prevented Staphylococcus aureus outgrowth in the cadaver mouse bone-defect model in bone and surrounding soft tissue. Bars indicate mean +SD, n = 3 per group. Dotted line represents bacterial inoculum.

The cadaver mouse bone-defect model was used to evaluate the applicability and bactericidal activity of BAG cream in a complex tissue environment. During application, the BAG cream demonstrated suitable handling characteristics: it was smoothly administered using a syringe and remained localized at the defect site indicating adequate adherence to both bone and surrounding tissue. The consistency of the formulation allowed uniform coverage of the defect, indicating potential applicability for use in irregular bone cavities or wound like settings. The bactericidal efficacy of BAG cream was assessed after inoculation of the defect with *S. aureus* in no-treatment (control) and treatment groups (BAG cream). After overnight incubation a 2-log increase in bacterial numbers was recorded in the no-treatment group relative to the challenge dose (2.5 × 10^4^ CFU), whereas BAG cream prevented bacterial outgrowth in the treatment group (Figure 7).

## 4 Discussion

In this study we investigated a novel Bioactive Glass S53P4 cream for antimicrobial activity and ease of handling and application. We studied the broad-spectrum microbicidal activity, the relationship with released concentrations of silicon, sodium, calcium and phosphorous and with pH, and the biofilm killing activities of the cream and its eluates, focusing on their potential to help prevent and treat orthopedic implant-associated infections (OIAIs). Both BAG cream and powder eluates completely eradicated planktonic *S. aureus*, outperforming conventional bioactive glass granules and their eluates. BAG cream and its eluate exhibits bactericidal activity in the absence and in the presence of TAN implant materials. Elemental analysis of the BAG cream and powder eluates revealed that levels of silicon and sodium increased while calcium and phosphorous levels decreased, when compared to eluates of binder. The BAG cream and powder eluates had alkaline pH and exhibited complete killing of *S. aureus*. Notably, BAG cream and powder eluates collected after just 2 h exhibited strong broad-spectrum activity, including activity against multidrug resistant bacteria of the ESKAPE(E) panel, against the anaerobic pathogen *C. acnes*, and against the fungal species *C. albicans* and *C. auris*. Time-kill assay using 2-h BAG cream eluate revealed a rapid antibacterial effect, with significant reduction in bacterial numbers observed within 30 min and continued killing over time. Furthermore, both cream and powder eluates effectively killed *S. aureus* biofilms. The cadaver mouse bone-defect model allowed us to test the cream’s applicability in a complex environment and showed its ability to prevent *S. aureus* outgrowth in a localized bone defect.

Evaluating biomaterials such as BAG for their antimicrobial activity is often performed exclusively in direct contact with the target bacteria. However, in implant-associated infections bacteria may spread from the infected implant into the surrounding tissue (Broekhuizen et al., 2007; de Breij et al., 2018; Jensen et al., 2017; Riool et al., 2017). Therefore, eluate testing is important to assess the antimicrobial potential of released compounds. In our study, BAG cream and powder eluates obtained after only 2 h of elution eradicated a broad spectrum of multidrug resistant pathogens. Killing started very rapidly, within 30 min, showing the rapid action of BAG cream.

Our study showed that BAG powder and its eluates had stronger bactericidal activity than BAG granules and their eluates, likely due to the powder’s higher surface area-to-volume ratio, which enhances ion release, a finding also supported by Zhang et al. (Zhang et al., 2010). Eluates of BAG cream and BAG powder showed similar bactericidal and biofilm-killing effects, indicating that the binder in the cream does not affect the bactericidal activity. Despite similar bactericidal activity, Si, Na and Ca levels were slightly lower in cream eluates compared to powder eluates. This suggests that the binder in the cream likely modulates the ion concentration rather than significantly hindering it. Interestingly, in direct testing the binder alone caused a 1-log bacterial reduction, likely due to its constituents PEG and glycerol, components known for mild antibacterial activity (Nalawade et al., 2015; Saegeman et al., 2008). This added effect may help prevent early-stage infections, enhancing the overall efficacy of the BAG cream.

Titanium and its alloys are widely used in orthopedic implants for their strength, biocompatibility and corrosion resistance, but remain prone to bacterial colonization and biofilm formation (Liang J et al., 2023). Our study shows that BAG cream effectively prevents bacterial colonization on titanium surfaces. TAN discs alone also partially reduced bacterial numbers, possibly due to release of titanium ions from the oxide layer (Liang Y et al., 2023). The relative contribution of this “TAN effect” to the full eradication achieved when cream was applied to the TAN discs is difficult to assess. In any case, our findings clearly show the potential of combining BAG cream with titanium implants to prevent bacterial colonization, enhancing infection control.

The 24-h elemental concentration profiles of the eluates of BAG cream and powder formulations indicate sustained reactivity of the glass with the surrounding liquid throughout the entire elution period, despite the relatively high glass surface area-to-volume ratio allowing quicker dissolution and saturation. This continued reactivity is likely maintained by the high pH of the eluates, which arises from the initial release of ions from the glass. The high pH promotes Si solubility and prevents glass dissolution from being halted by Si saturation (Lopez-Fontal and Gin, 2025). Sodium ions, as glass network-modifying ions, are loosely bound within the relatively open silicate structure of BAG S53P4 and readily dissolve into the surrounding solution. Therefore, the (magnitude of the) increase in sodium concentration relative to the RPMI medium serves as a reliable indicator of the fraction of original glass that has reacted. The rapid increase in sodium concentration within 2 hours, followed by a more gradual rise reflects an initial burst of ion release from the glass surface. Thereafter sodium levels rose more gradually. This indicates that glass reactivity persisted at a lower rate. Similar trends have been reported previously (Aalto-Setälä et al., 2023). The early depletion of calcium and phosphorous relative to the RPMI controls suggest rapid supersaturation and subsequent precipitation of calcium phosphate phases, a known step in the bioactive glass reaction sequence that precedes apatite-like layer formation (Horkavcová et al., 2025). A pronounced decrease in Si concentration between 8 and 24 h likely reflects secondary precipitation processes such as condensation and polymerization of dissolved Si species into colloidal or gel-like phases. The eluates consistently contained disproportionately higher Na relative to Si than expected for uniform (congruent) dissolution. The pattern aligns with preferential leaching of network-modifying ions such as Na and reprecipitation of dissolved Si as solid gel phases, as reported for S53P4 and related compositions (Siekkinen et al., 2022). Overall, these findings indicate that BAG S53P4 dissolution is governed by two concurrent processes: continuous ion release (like Na) from the glass network and localized precipitation of calcium phosphate and Si phases. Together, these dynamic physiochemical changes shape the solution environment and are likely to influence the antibacterial performance of the BAG.

While the results show that elevated pH is necessary for the antibacterial effect of BAG S53P4, pH alone does not fully explain the bactericidal activity observed. BAG cream, powder and granule eluates all had high pH levels, whereas at lower amounts of BAG only the BAG cream and powder eluates had bactericidal activity. This disparity likely arose from differences in ion release kinetics linked to surface area-to-volume ratio. Thus, the antibacterial activity of BAG S53P4 depends not only on alkalinity but also on the combined effect of bioactive ions released during glass dissolution, warranting further investigation to identify which ions are involved and elucidate their precise roles in the microbicidal mechanism. Biofilms are difficult to eradicate due to their protective extracellular matrix and antibiotic-tolerant microbial cells, posing significant challenges in revision surgeries (Gbejuade et al., 2015). Bioactive glass S53P4 and 45S5 particles (32–125 µm) have demonstrated *in vitro* efficacy in reducing *S. aureus* biofilms through ion release and pH modulation (Zhou et al., 2022). In line with this, a longer elution period for our BAG cream and powder eluates correlated with increase in their biofilm killing activity and continued glass dissolution. The eluate of binder alone exhibited significant biofilm killing activity at 4 and 8 h of elution. The binder contains glycerol which acts as a mild surfactant that alters the biofilm matrix and affects bacterial viability, without exerting strong bactericidal effects against planktonic cells (Fu et al., 2021). Notably, ICP-OES analysis of BAG cream and powder eluates showed a decline in silicon concentrations at later time points, likely reflecting Si precipitation. This observation suggests a potential combined mechanism: i) precipitated Si particles may disrupt the biofilm matrix increasing bacterial exposure (Devlin et al., 2021) and ii) dispersed colloidal silica generated during the glass dissolution could contribute to oxidative stress within the biofilm (Hetrick et al., 2009). Togetherwith the alkaline environment, these processes may underlie the enhanced biofilm-killing observed. These distinct temporal dissolution processes of rapid rise in ions, sufficient to kill planktonic cells combined with prolonged chemical stress essential for killing bacteria in biofilms, highlight the therapeutic relevance of prolonged exposure to BAG dissolution products for maximizing biofilm killing potential.

The cadaver mouse bone-defect model was used to assess the practicality and antibacterial efficacy of the BAG cream. The formulation’s injectability enabled precise placement at the defect site, where it effectively adhered to both bone and adjacent tissues. Its viscosity and consistency promoted thorough defect coverage, demonstrating its suitability for application in anatomically irregular bone cavities or complex wound environments. These handling characteristics are essential for potential clinical translation in managing challenging bone infections and defects. While BAG cream prevented bacterial outgrowth in bone and surrounding tissue, small numbers of bacteria remained. The cadaver model however is only a rough estimate of the conditions in live animals, since there is no active immune system nor effective antibiotic prophylaxis. In a live rat non-union bone infection model, BAG S53P4 putty alone also was insufficient to clear infection when systemic antibiotics were not given (Freischmidt et al., 2022). In contrast, combining borosilicated BAG-PMMA cement with low-dose gentamicin in a rat bone implant infection model did significantly enhance bacterial clearance (Fan et al., 2025). These findings suggest that effective infection control in the complex bone environment requires more than BAG alone. In clinical practice, local administration of BAG S53P4 granules or BAG putty is always combined with systemic antibiotic treatment and this combination has shown efficacy in one stage revision surgery of infected orthopedic implants (Gatti et al., 2024; Ziegenhain et al., 2021). A retrospective study on mastoid and epitympanic obliteration also supports the importance of adjunct antibiotic therapy, since a lower infection rate was observed when BAG granules were combined with antibiotics (4% infection) compared with their use without antibiotics (22% infection) (Alciato et al., 2022). This suggests the need to combine BAG with antibiotics or other antimicrobials in clinical practice and indicates that the BAG S53P4 cream described here may serve as a suitable platform for such combination therapies due to its favorable handling characteristics and potent baseline antimicrobial activity.

Overall, this work supports BAG S53P4 cream as a promising agent for local antimicrobial strategy. Future studies should include *in vivo* evaluation to confirm efficacy, examine potential inflammatory responses to released ions under physiological conditions, explore synergistic BAG cream-antibiotic and antifungal combinations and validate long-term safety and efficacy.

## Data Availability

The original contributions presented in the study are included in the article/Supplementary Material, further inquiries can be directed to the corresponding authors.
