# Supplementary material for: Bioactive glass S53P4 cream kills ESKAPE panel multidrug resistant pathogens and Staphylococcus aureus biofilms
Source: Front Pharmacol. 2026 May 4;17:1768338. doi: 10.3389/fphar.2026.1768338 (PMC13181238; doi:10.3389/fphar.2026.1768338)
Supplement: Supplementary file 1 [file DataSheet1.pdf]

Supplementary Material

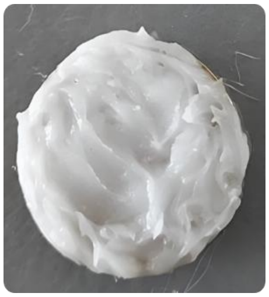

Figure S1: The figure depicts BAG cream consists of 50% powder (<25 µm) in weight and 50% binder applied on TAN disc

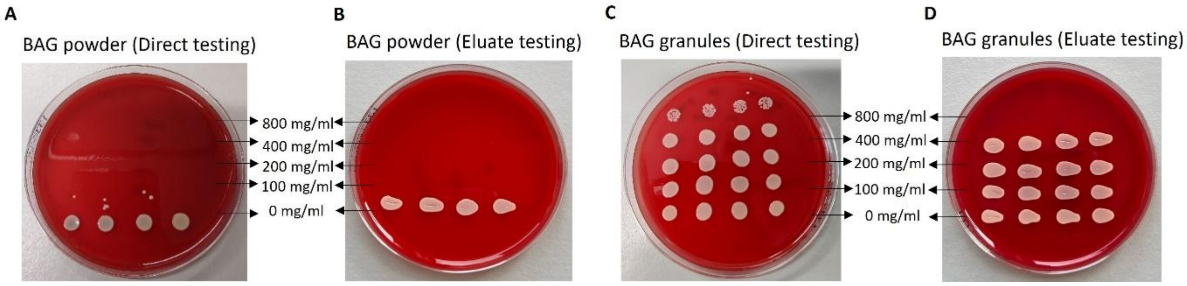

Figure S2: Bactericidal activity of BAG powder and granules in direct and eluate testing after 24 hours exposure to *S. aureus*. Blood agar images showing *S. aureus* growth, spotted undiluted in fourfold, after exposure to different concentrations of (A) BAG powder, (B) BAG powder eluates, (C) BAG granules and (D) BAG granule eluates. Bacterial growth is shown as a spot with confluent bacterial growth or individual colonies. The experiment was performed in duplicate and representative images from one of the duplicate plates are shown.
